# Water Quality Improvements and Hydrogeomorphic Change Over 20 Years Contributed to Macrophyte Community Recovery in the Upper Mississippi River

**DOI:** 10.1111/gcb.71121

**Published:** 2026-10-06

**Authors:** Danelle M. Larson, Mirkka M. Jones, Benjamin Weigel, Brian Gray, Otso Ovaskainen

**Affiliations:** ^1^ U.S. Geological Survey La Crosse Wisconsin USA; ^2^ Research Centre for Ecological Change, Organismal and Evolutionary Biology Research Programme University of Helsinki Helsinki Finland; ^3^ Institute of Biotechnology, HILIFE Helsinki Institute of Life Science University of Helsinki Helsinki Finland; ^4^ INRAE, EABX Cestas France; ^5^ Department of Biological and Environmental Science University of Jyväskylä Jyväskylä Finland

**Keywords:** aquatic plants, aquatic vegetation, hierarchical modelling of species communities (HMSC), re‐oligotrophication, river restoration, Upper Mississippi River, water quality

## Abstract

Several large rivers across the globe have recently undergone noticeable improvements to water quality. Yet, the cascading ecological consequences for aquatic life, including macrophytes, are largely unknown, owing to the lack of suitable long‐term data and the difficulty of modelling community‐level responses. Our study leveraged multiple big datasets from 1998 to 2019 within a 400‐km stretch of the Upper Mississippi River in the United States, which experienced major changes to hydrogeomorphology, water quality, and climate during the 22‐year study. These decadal changes resulted from both natural processes, such as increased discharge and hydrologic connectivity, and targeted management actions, including water quality improvements and restorations. Using joint species distribution models, we showed how changes to hydrogeomorphology and water quality increased macrophyte taxonomic and ecological (trait) diversity at a macroscale. We uncovered substantial macrophyte community recovery as evidenced by increased species prevalence and diversity, as well as favorable community trait shifts from undesirable free‐floating plants towards desirable submersed plants. Submersed species were positively associated with water quality improvements (specifically, reduced concentrations of total phosphorus, chlorophyll *a*, and total suspended solids). However, total nitrogen concentration modestly increased over the 22‐year period and nearly all macrophyte species were locally absent or negatively affected by high nitrogen values. Pollination mode and life form traits responded to environmental changes; as examples, submersed plants recovered with water quality improvements and epihydrophilous plants that pollinate on the water's surface increased with hydrogeomorphic connectivity. Water quality improvements explained approximately 25%–40% of the macrophyte community's temporal variation. Other major environmental drivers of macrophyte distribution included spatial variations of water depth and velocity. Macrophyte community assembly was shaped partially by strong temporal changes in hydrogeomorphology and water quality, and to a greater extent the spatial habitat heterogeneity within the large riverscape.

## Graphical abstract

Re‐oligotrophication drove strong recovery of macrophyte communities in the Upper Mississippi River, increasing plant diversity and shifting dominance toward submersed species. This ecosystem‐wide transition signals biological recovery from improved water quality.

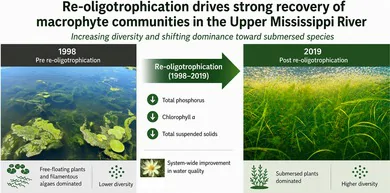

## 1 Introduction

Globally, rivers reveal a striking contrast: while a few systems show signs of ecological recovery in the early 21st century (Diamond et al. 2022; Houser 2022; Ibáñez et al. 2023), most continue to degrade under intensifying human pressures (Feio et al. 2023). Well known drivers of degradation include altered hydrology, nutrient and sediment enrichment, and climatic changes that can trigger cascading losses in freshwater biodiversity and ecosystem services (Datry et al. 2018; Huang et al. 2019; Sparks et al. 1998). Yet, the processes that enable some rivers to rebound following degradation remain poorly understood and limit the development of effective strategies for restoration and policy under accelerating global change. To address this gap, we leveraged decades of ecological data and community modelling from the Upper Mississippi River (UMR) in the United States (US), which is a rare example of a large river worldwide with an extensive monitoring record showing recovery of documented water quality improvements and macrophyte resurgence.

The UMR provides an exceptional opportunity to investigate recovery mechanisms because its trajectory toward renaturalisation reflects the combined influence of large‐scale restorations, extensive long‐term monitoring, and natural processes that were amenable to recovery. The US federally funded Upper Mississippi River Restoration (UMRR) program and its Long Term Resource Monitoring (LTRM) element have generated one of the most comprehensive ecological datasets for any large river that supports assessment of water quality, biological communities, and habitat changes (Houser 2022). These data not only document the UMR's progress toward recovery but also inform restoration planning, adaptive management, and national policy aimed at improving river health (e.g., McCain et al. 2018; Upper Mississippi River Basin Association 2026).

The LTRM revealed that the UMR has undergone substantial ecosystem change over recent decades, reflecting both natural processes and intense restoration actions (Houser 2022). These changes vary somewhat among river reaches due to differences in tributary inputs, hydrologic connectivity, and restoration intensity, but several broad trends are clear. Upper reaches continually experienced greater floodplain inundation and hydrologic connectivity over many decades due to more precipitation and total discharge (Van Appledorn et al. 2026). Water quality monitoring showed marked declines in the upper reaches for total suspended solids (TSS), total phosphorus (TP), and chlorophyll *a*, which signals improving water quality (Davis et al. 2025; Jankowski 2022). The major reasons for water quality improvements in the UMR included (Jankowski 2022): (1) implementation of US Clean Water Act regulations over several decades, such as upgrades to metropolitan water treatment plants before discharging nutrient‐rich wastewater into the Mississippi River, (2) reduced TP inputs from major tributaries, and (3) reduced TSS from decreases in agricultural run‐off and massive river‐island restorations that prevent erosion. Simultaneously some stressors remain, including persistent total nitrogen (TN) enrichment and episodic exceedances of TSS and TP regulatory thresholds. Concurrently, macrophytes recovered as water quality improved, although the environmental drivers of this resurgence are marginally understood (Burdis et al. 2020; Bouska et al. 2022; Larson et al. 2022). These dramatic physical and chemical changes likely drove macrophyte recovery in the UMR and mirror global patterns in other large rivers, offering insight into why some ecosystems recover while others do not.

Macrophyte rebound was recently documented in several other large rivers worldwide, placing the UMR within a broader pattern of resurgence. Notable examples include the lower Ebro River in Spain (Ibáñez et al. 2012), River Spree in Germany (Ibáñez et al. 2023), Loire River in France (Diamond et al. 2022), River Rhine in northwestern European countries (ICPR 2015) and the UMR in the US (Houser 2022; Larson, Carhart, and Lund 2023). These recoveries are presumably associated with “re‐oligotrophy,” i.e., notable water quality improvements from the reductions in nutrients and suspended sediments (Davis et al. 2025; Ibáñez et al. 2023). The water quality improvements can shift dominance away from fast‐growing, free‐floating plants and algae toward more stable and diverse macrophyte communities. Free‐floating plant dominance can reduce habitat quality and elevate the risk of harmful algal blooms that cause injury to people, wildlife, and economies (Igwaran et al. 2024). In contrast, macrophyte‐dominated systems support high quality waterfowl and fish habitats and are frequently targeted in restoration programs, including in the UMR (McCain et al. 2018) and its regional shallow lakes (Larson et al. 2020). While conceptual models provide insight into how macrophyte assemblages may shift under change (Hilton et al. 2006; O'Hare et al. 2018), quantitative assessments remain limited because long‐term data are rare and the interacting drivers of recovery are difficult to disentangle (Alahuhta et al. 2021). These knowledge gaps highlight the need for mechanistic community‐level approaches capable of resolving the ecological and physical processes shaping macrophyte assembly during recovery.

Community assembly theory and models help elucidate the many facets and environmental drivers of biodiversity (Ovaskainen et al. 2017; Belitz et al. 2025). Macrophyte communities likely reflect interactions among environmental filtering and niche partitioning (Bouska et al. 2022; Larson et al. 2022), biotic interactions (LeBagousse‐Pinguet et al. 2012), dispersal constraints (Correia Nunes et al. 2021), and species traits and evolutionary relationships that are influenced by stochastic processes (Dalla Vecchia et al. 2020; Fu et al. 2020; García‐Girón et al. 2019). Joint species distribution models, such as the Bayesian Hierarchical Modelling of Species Communities (HMSC) framework, can integrate community assembly processes with the spatiotemporal dynamics across nested, hierarchical scales (Ovaskainen et al. 2017; Tikhonov et al. 2020; Weigel et al. 2023). Understanding the relative roles of each assembly process, especially spatial and temporal processes shaped by global changes, can advance macrophyte ecology (Alahuhta et al. 2021) and guide global river restorations.

In this study we sought to understand how two decades of hydrogeomorphic, water quality, and climatic changes in the UMR affected macrophyte taxonomic and functional (ecological trait) diversity across multiple spatial scales, including the macroscale covering 400 river kilometers (km). We applied HMSC to 22 years of monitoring data in four river reaches to identify the processes associated with the macrophyte recovery previously documented in the UMR (Bouska et al. 2022; Larson, Carhart, and Lund 2023). Our study was guided by three expectations. First, we expected that water quality improvements would promote diversity and shifts in ecological traits, particularly a transition from dominance by free‐floating plants toward a more diverse, submersed plant community. Second, we expected epihydrophilous plants (those that pollinate on the water's surface) to benefit from increased water due to their water dispersal and reproductive strategies (Kim and Nishihiro 2020). Third, we hypothesized that macrophytes would respond positively to hydrogeomorphic connectivity, which provides abundant shallow habitat across the unconfined floodplain. To evaluate these expectations, we used HMSC to quantify long‐term shifts from free‐floating plant dominance to submersed macrophyte diversity and identify the key spatial and temporal environmental drivers of community change.

## 2 Material and Methods

### 2.1 Study Area

The UMR is approximately 2200 river km in length in the US (Figure 1). A total of 29 locks and dams have been operating since their installation in the early 20th century. These locks and dams divide the river into navigation pools or reaches that allow barge traffic on the main channel throughout the ice‐free months. Each reach is composed of multiple sampling strata, including main channel borders, side channels, contiguous floodplain lakes, and contiguous impounded areas upstream from the dams. Channel borders are areas along the main channel that may support species that can withstand deeper waters and fast velocity. Side channels are hydrologic extensions of the main channel with unidirectional flow. Contiguous floodplain lakes have varying degrees of water depth, flow, and connectivity to the main channel. Contiguous impounded areas are open and expansive shallow waters (less than [<]1 meter [m] deep) upstream from locks and dams. Historically, the newly installed locks and dams created expansive aquatic areas with stabilized water levels that allowed macrophytes to flourish for decades (Moore et al. 2010).

**FIGURE 1:**
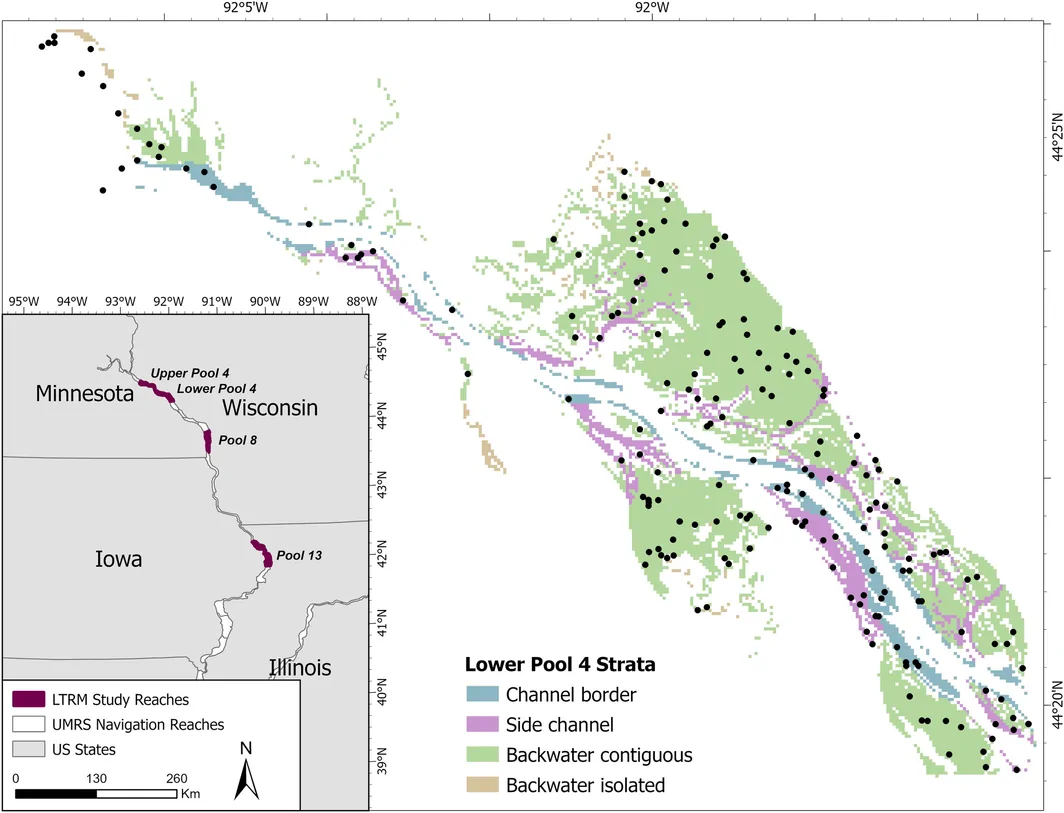
The inset map of the UMR showing four study reaches with long‐term resource monitoring (LTRM) included in this study (“LTRM Study Reaches”), which span ~400 river km. Using Lower Pool 4 as an example, the primary map displays the four strata (in colors) and examples of macrophyte sampling plots (black dots) for a given year selected via a stratified random sampling design. The white areas are unsampled for aquatic plants because the plots are on land or the main channel, which cannot support macrophytes.

A sudden, system‐wide macrophyte decline occurred in the late 1980s for reasons undocumented but likely driven by high nutrient and sediment loads, drought, and island erosion (Fischer and Claflin 1995; Rogers 1994). The UMRR program was initiated in the early 1990s to conduct ecological science and habitat restoration for which restoring macrophyte abundance and diversity is one of several objectives (McCain et al. 2018). This study used LTRM data from 1998 to 2019 to assess relationships of hydrogeomorphology, water quality, and climate with the macrophyte recovery that occurred during a time of great ecosystem change during the 2000s (Houser 2022; Larson et al. 2022).

### 2.2 Sampling Design and Environmental Covariates

The UMRR program has monitored aquatic macrophytes, water quality, and hydrogeomorphology for decades using standardized protocols and a stratified random sampling design (De Jager et al. 2018; Larson et al. 2025; Soballe and Fischer 2004). We included four study reaches named Upper Pool 4 (~19 km), Lower Pool 4 (~19 km), Pool 8 (~37 km), and Pool 13 (~54 km; Figure 1). These reaches have multiple environmental gradients and marginally different macrophyte community composition (Larson, Carhart, and Lund 2023).

All macrophyte species were surveyed annually during summer using a standardized protocol that included 450 sampling units (plots) per reach in Pools 4, 8, and 13 (Larson et al. 2025). Sampling plots were selected under a stratified random sampling design, with the four strata corresponding to previously described hydrogeomorphic features: main channel borders, side channels, contiguous floodplain lakes, and contiguous impounded areas. Given randomization and relatively low sampling probabilities, units were effectively never resampled (an exception was that units in Pool 8 were intentionally resampled from 2000 to 2003). We split Pool 4 into two reaches after stratification (yielding ~225 units each year that we named Upper and Lower Pools 4) because the large and influential Lake Pepin (~35 km) separates these two reaches and causes distinct habitat characteristics, macrophyte and fish assemblages, and differing degrees of water quality improvements between upstream (Upper Pool 4) and downstream (Lower Pool 4) of Lake Pepin (Burdis et al. 2020; Larson et al. 2022). Units with predicted water depths > 2.5 m under standard discharge were excluded from the sampling frame because macrophytes are difficult to sample effectively in deep, fast‐flowing water (Larson et al. 2025) and because macrophytes in this turbid river are known to typically be absent at depths exceeding 2.5 m (Delaney and Larson 2024).

At each plot, macrophyte experts sampled the macrophyte community using procedures detailed in Larson et al. (2025). The macrophyte sampling plot was defined as a 2‐m rectangular area around the sampling boat (plot area is ~44 square meters). All macrophyte species and life forms were recorded within the plot using visual assessments for species detections and percentage of cover. All submersed species were also recorded using a modified garden rake at six locations around the boat, and each species was given an ordinal biomass score from 0 to 5. Most species have moderate to high detection probabilities (Gray 2021). In total, the LTRM experts sampled ~30,000 macrophyte plots over 22 years among the four reaches.

Environmental covariates were chosen by consulting scientists and resource managers at a macrophyte restoration workshop hosted by author D. M. Larson in 2021. Using workshop participants' knowledge and literature, we initially considered 22 environmental variables deemed most important for macrophyte occurrence; however, we ultimately chose nine variables for parsimonious modelling (Table 1). The variables represented three covariate groupings (hydrogeomorphology, water quality, and climate) and limited multicollinearity (*R* < 0.7 and variance inflation factor < 2). We used interpolation with a prediction accuracy > 93% following procedures by Larson, Bungula, et al. (2023) to fill in missing values for the covariates of velocity, total nitrogen (log transformed; logN), and total phosphorus (log transformed; logP).

**TABLE 1:** The relevance of nine covariates as environmental drivers of the aquatic macrophyte communities on the UMR. Hypotheses are those of the authors with important input from many Mississippi River stakeholders and regional macrophyte experts at a 2021 macrophyte restoration workshop. The public data sources are provided in table footnotes and References section.

Covariate grouping	Covariate acronym	Covariate (measurement units)	Relevance for macrophytes
Hydrogeomorphology	Depth^a^	Average water depth (m) at plot at time of macrophyte sampling	Determines the dominant plant life form Metric of habitat heterogeneity at multiple spatiotemporal scales Limiting for submersed plants due to light interception that affects photosynthesis
Hydrogeomorphology	Connectivity^b^, ^c^	Index of hydrologic connectivity (%). Calculated as the percent of perimeter from a 50‐m radius buffer around the plot that is aquatic.	Influences local hydrology and flow patterns, wind fetch, water quality, and macrophyte dispersal Metric of habitat heterogeneity, floodplain complexity, and human alterations to geomorphology Hypothesis: epihydrophilous plants (water surface pollinators) will benefit from greater connectivity that aids in dispersal (Kim and Nishihiro 2020)
Hydrogeomorphology	Velocity^c^, ^d^	Water velocity (meters per second)	Causes mechanical stress Limiting factor in river environments
Hydrogeomorphology	Exceedance^b^, ^c^	Water depth (centimeters) during low‐flow growing season conditions, representing the channel bathymetry at a discharge that is exceeded 75% of the time over the past 40 years. This is low‐flow depths tied to a Q75 exceedance threshold.	Signifies low water conditions that can stimulate emergent reproduction and increase water clarity by consolidating TSS Hypothesis: low water during summer can be a catalyst for plant re‐colonization and sustained emergent abundance (Lund 2019; Burdis et al. 2020; Delaney and Larson 2024)
Water quality	LogN and logP^d^, ^e^	Concentrations of total nitrogen and total phosphorus (mg/L), log‐transformed prior to modelling	Provides essential macronutrients needed for plant growth and reproduction High nutrient status indicates eutrophy Hypothesis: nutrient reduction supports macrophyte recovery due to a complex interplay of life forms competing and other possible mechanisms
Water quality	TSS^e^	Total suspended solids (mg/L)	Exponentially decreases light availability for photosynthesis. Excess TSS arises from agricultural runoff, sewage inputs, or shoreline and island erosion. Hypothesis: TSS reductions facilitate macrophyte recovery by improving water clarity needed for photosynthesis
Water quality	Chlorophyll^e^	Chlorophyll *a* concentration (μg/L); proxy for phytoplankton biomass	Phytoplankton are superior competitors to macrophytes for nutrients and light High chlorophyll *a* indicates eutrophic waters Hypothesis: chlorophyll *a* reduction supports submersed macrophyte recovery by reducing competition for nutrients and light
Climate	Temperature^e^	Water temperature (°C)	Affects macrophyte metabolic rates, reproductive cycles, nutrient leaching, and decay rates Causes mortality to heat‐intolerant species

^a^ Long‐Term Resource Monitoring Program—SRS Vegetation Data Query (usgs.gov).

^b^ Rohweder (2018) (https://doi.org/10.5066/F7Z89BMP).

^c^ De Jager et al. (2018) (https://doi.org/10.3133/ofr20181143).

^d^ Missing data were interpolated following procedures from Larson, Bungula, et al. (2023).

^e^ UMESC—Long Term Resource Monitoring element—Water Quality.

High data densities allowed data to be integrated at the macrophyte plot‐level. We used ArcGIS version 10.7.1 to merge the environmental covariates (Table 1) that were closest in space and time to each macrophyte plot. The “cost distance function” within ArcGIS ensured that the nearest water quality sample and hydrogeomorphic variables were hydrologically connected to the macrophyte plot without land barriers. We further extracted water quality values recorded within < 60 days and < 600 m of each macrophyte plot.

We calculated unique, plot‐level values for the hydrogeomorphic covariates named “connectivity” and “exceedance” using ArcGIS version 10.7.1. Hydrogeomorphic connectivity was defined as the percentage of the 50 m buffer perimeter (around each plot) that was aquatic, using a static land/water coverage derived from 2010/2020 land cover data (USACE 2017). Exceedance describes low water depth during the growing season and was calculated as the mean pixel value within a 50 m buffer surrounding each plot using a static bathymetry layer corresponding to the 75th exceedance probability for water levels at the upstream lock and dam. The temporal variation (as annual covariate means) for both metrics had two sources: (1) sampling error associated with the stratified random sampling design, which depends on which plots within each stratum are selected at random each year, and (2) changes in the annual covariate means at vegetated plots that signify how the vegetated plot conditions changed annually. Temporal changes in both metrics reflect changes in the distribution of vegetated sampling plots because the spatial data layers they are calculated from did not change over time. Water temperature from the LTRM dataset was selected for inclusion in our statistical models as representative of climate. We initially extracted multiple air temperature and precipitation variables per plot (PRISM Climate Group 2021). Air temperature and water temperature were positively correlated (95%) and precipitation was highly correlated with exceedance (80%). Therefore, we included water temperature and not air temperature to represent climate. We acknowledge that hydrogeomorphic covariates are also influenced by climate, namely precipitation, and so we may underestimate the influences of climate by grouping connectivity and exceedance under hydrogeomorphology.

### 2.3 Macrophyte Community, Phylogeny, and Traits

To account for niche conservatism (i.e., the tendency for species to retain ancestral traits over evolutionary time, thus affecting species responses to environmental changes today), we included the taxonomic relationships of species in our modelling framework. All species are fully listed in Table S1. Because a phylogeny with branch lengths representing evolutionary divergence was not available for macrophyte species, we constructed a taxonomic tree as the proxy for species relatedness using the *as.phylo function* in the *ape* package (Paradis et al. 2020) in software *R* (R Core Team 2024), assuming equal branch lengths between the taxonomic levels of family, genus and species. Our models also evaluated to what degree traits affected species niches by including two ecological traits, life form (a morphological trait) and pollination mode (a life history trait). Life form included three levels: submersed, emersed, and free‐floating. Pollination mode included three levels: epihydrophily (above water), hypohydrophily (below water), and entomophily (insects). These traits were compiled in Table S1 from multiple sources (Devendorf 2013; Du and Wang 2014; Haynes 1988; Kim and Nishihiro 2020; Kleyer et al. 2008; U.S. Department of Agriculture 2022).

### 2.4 Data Analyses

All data, metadata, a workflow graphic, and analysis scripts are available from Larson, Rohweder, et al. (2026) and Larson, Jones, et al. (2026). Our analysis workflow was comprised of four major steps:

1. Regions of common profile: Classifying species composition into RCPs to explore macrophyte community compositional shifts through time, their impacts on different facets of biodiversity, and their ecological trait implications.
2. Partitioning environmental variation: Dividing each environmental covariate into two components to explore the relative roles of temporal and spatial/spatiotemporal environmental variability as drivers of macrophyte distributions.
3. Hierarchical community modelling: Fitting separate HMSC models for each of the four study reaches to quantify species responses to spatial and temporal variation in hydrogeomorphology, water quality, and climate, and to evaluate trait–environment relationships and taxonomic structuring of species responses. Model performance was assessed using multiple explanatory and predictive metrics.
4. Community‐weighted environmental trends: Calculating and plotting annual community‐weighted means (CWMs) of environmental covariates and fitting generalized linear models to characterize long‐term environmental trends associated with macrophyte distribution over the 22‐year study period.

Here we provide more details for each step.

#### 2.4.1 Step 1: Regions of Common Profile

To summarize macrophyte community change over the 22‐year period, we first calculated RCPs using (1) data from all reaches combined (~400 km) and (2) data from Lower Pool 4 only (~19 km). The RCPs from the 400 km reach provide a macroscale perspective, but combining those results with the HMSC models was computationally too intensive. Therefore, we also generated RCPs for Lower Pool 4 only, which adequately represents the other reaches in terms of community composition and environmental changes during the 22‐year study period. We highlight the Lower Pool 4 RCP results in the main text because of its ecological relevance and computational efficiency, but also provide the full multi‐reach RCP outputs in the S1.

Differences between RCPs are reported in terms of their species composition, species prevalence, diversity facets, and ecological traits, as well as the proportion of plots that shifted between RCPs over 22 years. We omitted plots with no detected vegetation (~20% of samples) before calculating pairwise Jaccard dissimilarities, focusing the analysis on compositional differences among plots with detected vegetation (hereafter “vegetated plots”). Although excluding unvegetated plots removes information about transitions from unvegetated to vegetated states, exclusion prevents zero‐inflation from dominating the dissimilarity matrix and allows clear inference about community composition and diversity change over time. To calculate the RCPs, we ran an ordination on the Jaccard matrix and included all ordination axes as the input for clustering. We used *k*‐means clustering on the ordination matrix, testing 1–10 clusters and selecting the optimal number using the Calinski criterion in the *vegan* R package (Oksanen et al. 2019). Then, each plot was assigned an RCP score and graphed over time to explore community shifts.

The annual mean species richness and ecological diversity (specifically, the functional dispersion [FDis] metric) were computed separately for each RCP based on the life form and pollination traits using the *FD* package in R (Laliberté et al. 2014). We also investigated temporal changes in the diversity metrics and CWM traits in the RCPs over time to test hypotheses regarding increases in diversity and spatiotemporal shifts of multiple traits.

#### 2.4.2 Step 2: Partitioning Environmental Variation

For each reach, we partitioned every environmental covariate into two components: (1) its annual sample mean, and (2) the within‐year spatial residual (the observed value minus that annual sample mean). This allowed us to distinguish macrophyte responses to broad, system‐wide interannual environmental changes from responses driven by local spatial conditions within a given year. The annual means describe how macrophytes responded to long‐term, reach‐scale environmental shifts (e.g., water quality improvements), whereas the spatial‐residual coefficients quantify responses to localized conditions at the plot‐level (e.g., water depth during sampling). Although fine‐scale spatial drivers such as water depth and velocity are documented in the UMR (Delaney and Larson 2024), accounting for this spatial variability within the HMSC models was necessary to isolate and evaluate longer‐term community shifts associated with improving water quality and increasing hydrogeomorphic connectivity. This explicit partitioning of temporal and spatial variation is a new approach within the HMSC framework and provides a useful way for examining community‐level dynamics.

#### 2.4.3 Step 3: Hierarchical Community Modelling

We built one joint species distribution model for each study reach using HMSC (Ovaskainen et al. 2017; Ovaskainen and Abrego 2020). Using HMSC allowed us to jointly model the entire macrophyte community while accounting for shared environmental responses, hierarchical spatial structure, residual species associations, and species traits to best understand community‐level responses to long‐term environmental changes. We modelled the four reaches separately because the reaches may vary compositionally along hydrologic, water quality, and climatic gradients (Larson et al. 2022), and to allow for complex interactions among environmental covariates within reaches. Upper Pool 4 and Lower Pool 4 included data from all the original plots that were vegetated (*n* = 2640 and 2839 plots, respectively). To reduce runtimes (which took over a week with high performance computers) of intensively sampled Pools 8 and 13, we randomly subsampled the data in these two reaches to include a total of 5000 plots in each. We fit full HMSC models for each reach with the package *Hmsc* 3.0.11 (Tikhonov et al. 2020) in R 4.4.1 (R Core Team 2024). We fit probit regression models with binary species‐specific detection at the plot‐level as the response variable. We excluded rare species with < 20 occurrences, retaining a total of 16–32 species per reach.

The four models included fixed and random effects. The fixed effects were the nine environmental covariates (Table 1) each divided into two components of variability: temporal (as annual means) and spatial (as annual residuals). To avoid overfitting, we did not include second order polynomial terms. The total nitrogen and phosphorus concentrations were log transformed (hereafter the covariates are named logN and logP) prior to modelling because taxa are likely to respond more strongly to a unit increase in nutrients when concentrations are low than when high. The three random effects represent plot, stratum, and year (temporally structured). Preliminary analyses did not find support for community‐level spatial autocorrelation in any reach. Hence, unexplained variation was modelled as unstructured random effects for plot and stratum. The contributions of each random effect, total spatial effect, and total temporal effect to the total explained community variance were assessed by variation partitioning (Ovaskainen et al. 2017).

We assumed the default prior distributions of HMSC (Ovaskainen and Abrego 2020). Posterior distributions were sampled with four Markov chain Monte Carlo (MCMC) chains, and each ran for 375,000 iterations, of which the first 125,000 per chain were treated as burn‐in. Samples were thinned by 1000 to yield 250 post burn‐in samples per chain to reduce autocorrelation, data storage needs, and computational burdens. Finally, we evaluated MCMC convergence through the effective sample size and potential scale reduction factors of all the model parameters (Gelman and Rubin 1992). We computed model explanatory power based on models fitted to all the data and model predictive power via fivefold cross validation using folds on the plots. Due to long runtimes for our large dataset, cross validations used only 10% of the iterations used to fit the full models. We reported the explanatory and predictive powers of the HMSC model with species‐specific Tjur *R* ^2^ and the area under the curve statistics (Ovaskainen and Abrego 2020). Tjur *R* ^2^ measures how well the model explains observed species presences and absences, with values near 0 indicating little explanatory power and values near 1 indicating strong explanatory power.

The HMSC models output multiple parameters that may aid ecological interpretations. The alpha (*α*) parameter describes temporal autocorrelation among residual species occurrences, after accounting for the environmental covariates. The beta (*β*) parameters show each species' predicted response to every environmental covariate, here parsed into their annual means and residuals. Positive *β* estimates indicate a positive ecological response of macrophytes to higher covariate values, either across years (temporal variation) or within years (spatial variation), while negative *β* estimates indicate a negative response. The gamma (*γ*) parameter measures trait‐environmental relationships, which may indicate advantages or disadvantages of the trait within certain environments. The rho (*ρ*) parameter measures whether the residual variance among the species' environmental responses is independent (~0) or fully structured by their taxonomic relationships (~1); thus, high phylogenetic correlation parameters indicate that related macrophyte species respond similarly to the environment, which helps predict responses by rare species and describes niche conservatism.

#### 2.4.4 Step 4: Community‐Weighted Environmental Trends

To summarize long‐term environmental changes experienced by the macrophyte community, we calculated annual community‐weighted means (CWMs) for each environmental covariate. CWMs were derived from presence–absence observations by averaging plot‐level environmental conditions across the macrophyte species occurring at each vegetated plot, giving each present species equal weight. Annual CWMs were then computed by averaging CWM values across all vegetated plots within each year. This approach provided a community‐linked description of temporal trends in environmental drivers, offering an intuitive context for interpreting the multivariate species–environment relationships estimated by the HMSC models.

## 3 Results

### 3.1 Model Performances and Taxonomic Signals

All four HMSC models (one per reach) yielded satisfactory performance that allows for good ecological inferences. The MCMC chain convergences were good, as indicated by potential scale reduction factors of all parameter estimates and upper credible intervals (CI) < 1.1. All four models had good fit and high discriminatory power (mean area under the curve = 0.93–0.98 and mean effective sample size = ~1000). The models explained community assembly reasonably well, as indicated by mean Tjur *R* ^2^ values of 0.31–0.35. Predictive performance based on fivefold cross validation was low but informative, with Tjur *R* ^2^ values of 0.11–0.20. Temporal autocorrelation in species occurrences (conditional on environmental covariates) was universal among all reaches, varying from 5 to 13 years (*α* = 5–13, probability [*α* 1 > 0] = 1). Strong taxonomic signals (*ρ* near 1) show closely‐related macrophyte species responded similarly to the environment in all four reaches (posterior mean *ρ* = 0.89–0.97; lower 95% CI *ρ* = 0.85–0.96; upper 95% CI *ρ* = 1.00), indicating taxonomic niche conservatism and generalizability of reach‐level results to the macroscale.

### 3.2 Strong Riverine Changes and Macrophyte Community Composition Responses

The river's dramatic environmental changes over two decades were accompanied by shifts in macrophyte community composition and increased abundance, as reflected in CWM trait patterns showing increasing dominance of many species adapted to deeper water, clearer water (from reduced TSS), and lower nutrient conditions (Figure 2). Simultaneous changes of the river environment and its macrophyte community assembly were similar across the four reaches. Hydrogeomorphic conditions and macrophytes naturally shifted toward deeper water and greater velocity in later years (Figure 2A–D). As hypothesized, water quality showed long‐term improvements that supported macrophyte recovery; specifically in Lower Pool 4, logP declined by 20%, chlorophyll by 70%, and TSS by 80% over the 22‐year period (Figure 2E–G). In contrast, logN increased by 8% although with high inter‐annual variability (Figure 2H). Water temperature was the only covariate that varied by reach, whereby mean summer water temperature in Upper‐ and Lower Pool 4 and Pool 8 decreased by 1.5°C (Figure 2I), but Pool 13 experienced a 1.5°C increase.

**FIGURE 2:**
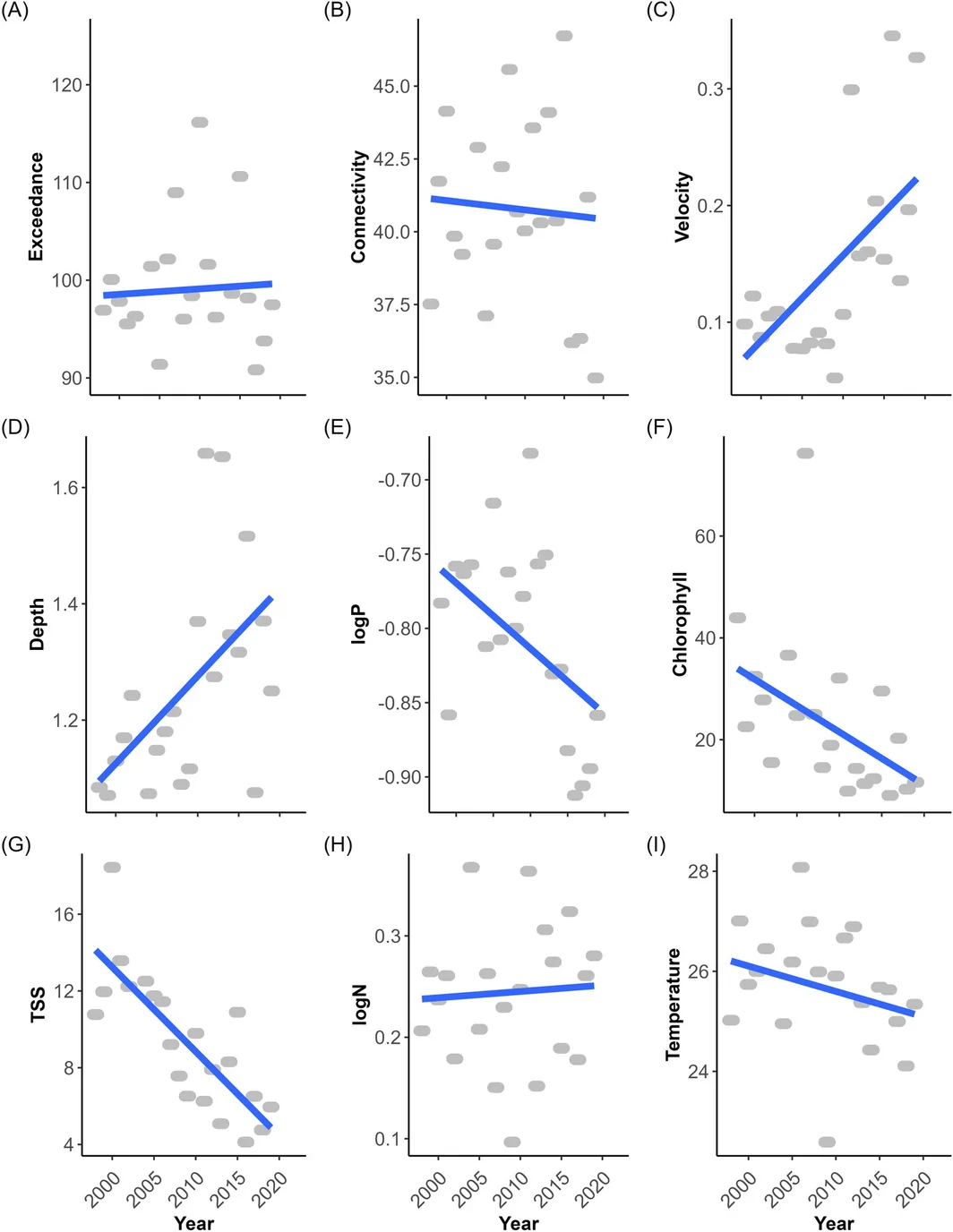
Annual community‐weighted means (CWMs) indicate long‐term, directional trends in the environmental conditions that correspond to macrophyte community change and recovery over the 22‐year study period. The CWMs (gray dots) are calculated from vegetated plots in Lower Pool 4 of Upper Mississippi River (*n* = 2839 plots) and the linear trends are shown as blue lines. The CWMs were calculated by weighting plot‐level environmental conditions by the relative prevalence of macrophyte species occurring at each plot, providing a community‐level summary of the environmental conditions experienced by dominant taxa. These aggregated indicators contextualize the multivariate species–environment relationships modelled in HMSC. These nine environmental covariates were hypothesized to affect macrophytes (Table 1): (A) exceedance, a measure of low water growing season conditions, calculated as water depth at 75% exceeded discharge over a 40 year history (cm); (B) connectivity, an index of hydrologic connectivity (0–100% connected); (C) water velocity (m/s); (D) plot water depth during time of macrophyte sampling (m); (E) total P concentration, log‐transformed (mg/L); (F) chlorophyll *a* concentration (μg/L); (G) total suspended solids (mg/L); (H) total N concentration, log‐transformed (mg/L); and (I) water temperature (°Celsius).

The evidence for macrophyte community composition shifts and recovery over the study period was strong and multifaceted in all reaches. The RCP clustering revealed two distinct groups based on species composition of 35 aquatic macrophytes (Figure S1). RCP1 was characterized by relatively low species prevalence, low species richness, and low ecological trait diversity of macrophytes compared to RCP2. Cosmopolitan taxa ( *Heteranthera dubia* [Jacq.] MacM., *Vallisneria americana* Michx., *Elodea canadensis* Michx., *Ceratophyllum demersum* L., and free‐floating plants) dominated RCP1, whereas RCP2 had 11 species with > 20% prevalence.

All reaches increased in macrophyte prevalence, diversity, and shifted species composition over 22 years (Figure 3). However, the timing, direction, nonlinearity, and the magnitude of such changes varied by reach (Figure S2). The diverse RCP2 increased strongly in extent in the early 2000s in Lower Pool 4 (Figure 3A), indicating a shift in community structure and greater macrophyte occurrences in later years. The RCP1 became more common after 2010 in Pools 8 and 13 due to an increase in cosmopolitan taxa and *Vallisneria americana* Michx., an ecologically important monoculture species. Taxonomic species richness increased for both RCPs over time (Figure 3B). The functional trait diversity (FDis metric) was much greater for RCP2 and low for RCP1 (Figure 3C). In support of our first hypothesis, the prevalence of submersed plants increased while free‐floating plant dominance was nearly gone by 2020 (Figure 3D,E). In support of the second hypothesis, epihydrophilous plants (that pollinate on the water's surface) increased by 20% for RCP1 (Figure 3F).

**FIGURE 3:**
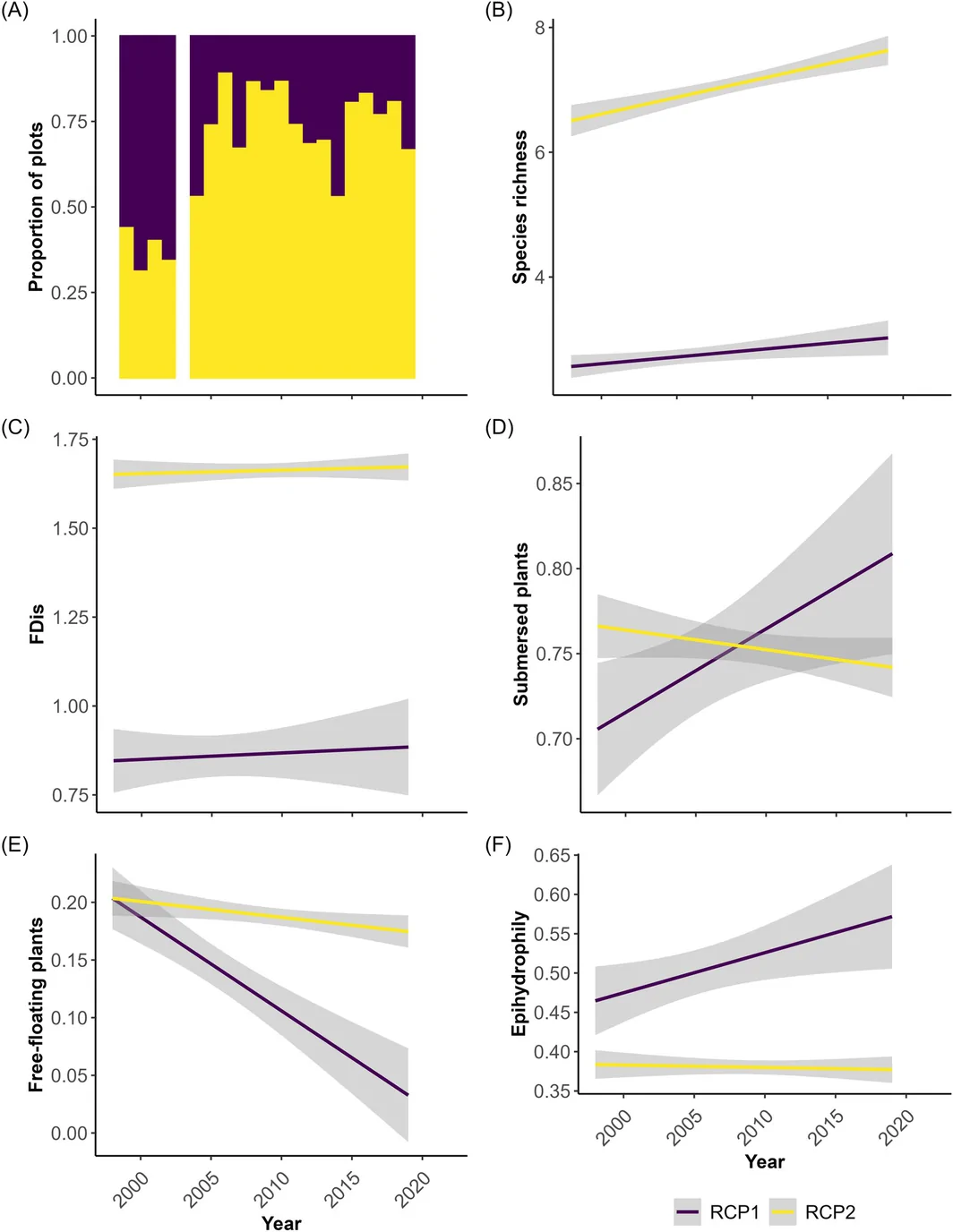
Time series of the relative extent of the taxonomic and functional trait changes from two macrophyte regions of common profile (RCP) from Lower Pool 4 of the UMR. Data was limited within Lower Pool 4 during 2003 and therefore are not shown. The trendlines denote annual mean values and the gray shading are 90% credible intervals. The RCPs were defined using *k*‐means partitioning and the Jaccard index with dissimilarity calculated for *n*=2028 vegetated plots. The *y* axes refer to (A) proportion of plots classified into each RCP each year; (B) species richness, or number of species; (C) functional diversity index (FDis) based on macrophyte traits; (D), prevalence of submersed plants; (E) prevalence of free‐floating plants; and (F) prevalence of epihydrophilous plants.

### 3.3 The Spatial and Temporal Drivers of Macrophyte Community Assembly

Macrophytes in all reaches were most responsive to spatial heterogeneity of the hydrogeomorphology covariates, followed by macroscale water quality improvements over time, and least responsive to spatial and temporal effects of climate (Figure 4). Of the explained variation, roughly 50% was due to spatial variation in local environmental covariates, 20% to macroscale temporal changes (like increased water and water quality improvements over 22 years), and 30% to the unexplained, random effects (mostly at the plot‐level). Climate, represented by water temperature only, explained < 1% of species occurrences despite the overall 1.5°C temperature decrease (Upper and Lower Pool 4 and Pool 8) or 1.5°C increase (Pool 13). Importantly in support of our hypotheses, the temporal variation from multiple water quality covariates collectively explained ~3%–20% of species occurrences, and their contribution to macrophyte recovery was greatest in Lower Pool 4 where the most water quality improvements occurred. Explained variation in species occurrences varied widely across species and reaches (4%–78%), leaving 22%–96% unexplained variation or noise. Variation (up to 70%) at the random effect of plot implies that some relevant environmental drivers are missing in our models or that species interactions are strong.

**FIGURE 4:**
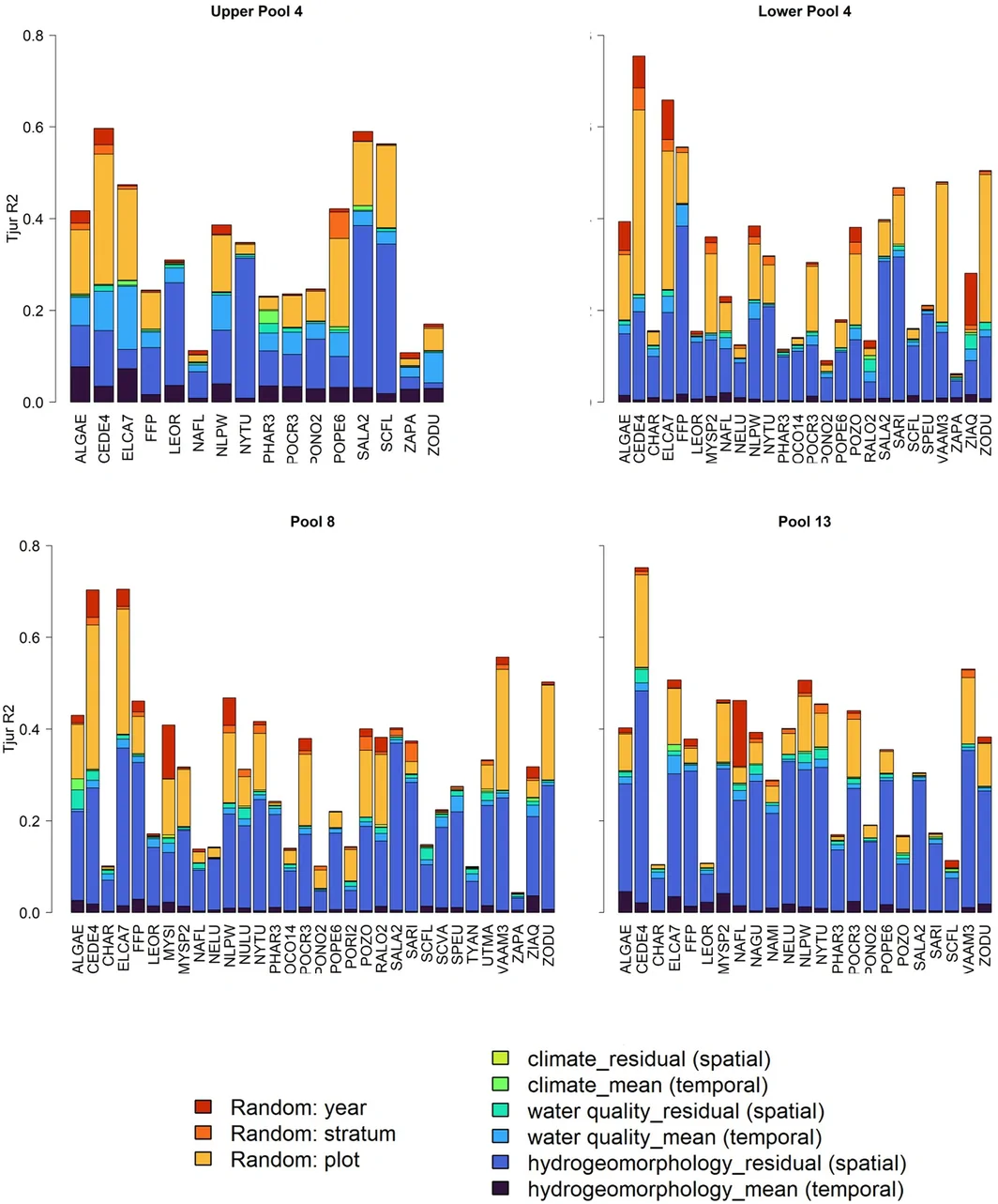
Stacked barchart shows variance partitioning (scaled by Tjur *R* ^2^) in macrophyte occurrence distributions explained by spatial and temporal processes at scales of the sampling plot, sampling stratum, and sampling year (represented by random effects, in reds and oranges) versus by the measured environmental covariates (hydrogeomorphology, water quality, and climate represented in violets, blues, and greens) in each of the four study reaches in the UMR. Each environmental covariate was represented by its annual mean (a temporal component of environmental variation) and its within‐year residual (a spatial component of environmental variation). Each bar shows variation explained for each macrophyte species. The species codes on the *x*‐axes are standards from the U.S. Department of Agriculture's Plants Database and detailed in Table S1. Table 1 lists details on each environmental covariate grouping.

The trait‐environmental associations often appeared strong in all reaches, particularly for the morphological trait of life form (Figure 5). The modelled traits of life form and pollination mode collectively explained 15%–40% of the total explained variation in species occurrences, depending on reach. Like the species‐environment relationships, trait variation was most responsive to hydrogeomorphology and water quality, but not to temperature.

**FIGURE 5:**
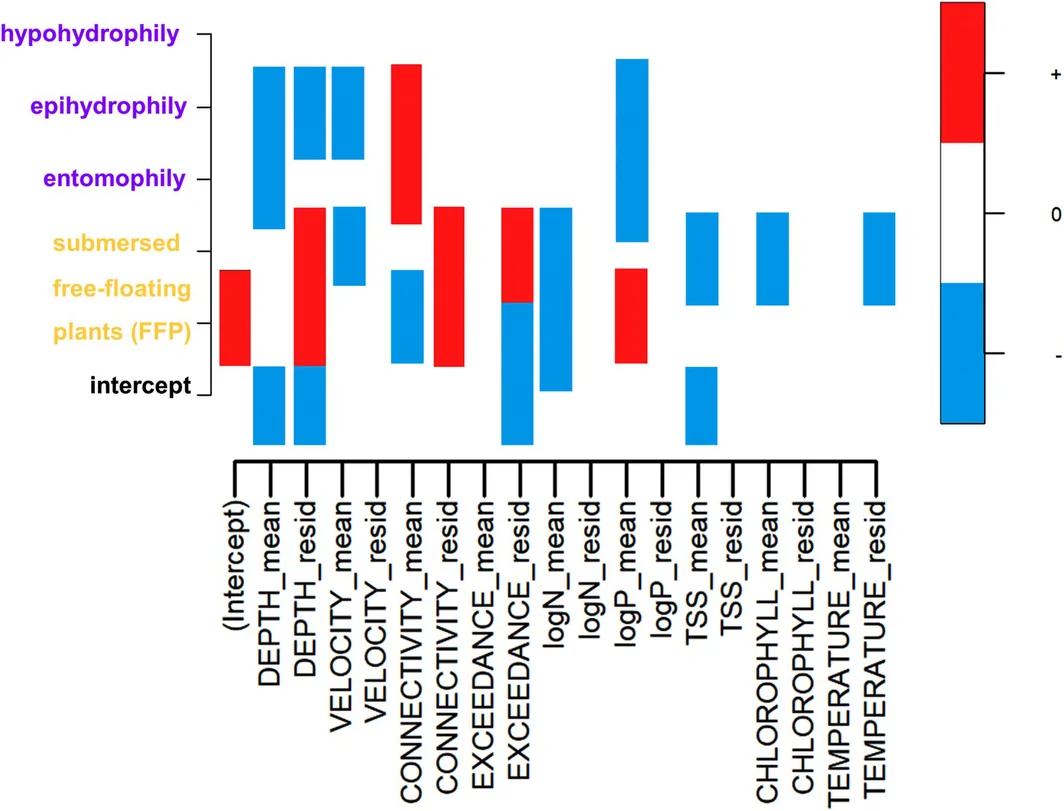
Macrophyte trait responses to covariates of hydrology, water quality, and climate combined from Upper Pool 4, Lower Pool 4, Pool 8 and Pool 13 of the UMR. The macrophyte trait‐environmental responses are separated into temporal and spatial components; specifically, covariate means (“COVARIATE_mean”) represent the temporal variation across 22 years and the residuals (“COVARIATE_resid”) represent the spatial variation within reaches and years. Positive and negative responses of each trait (> 90% posterior probability) are illustrated by the red and blue colors, respectively. Traits include life form (yellow; levels of free‐floating plants (FFP) and submersed) and pollination mode (violet; levels of hypohydrophily, epihydrophily, and entomophily).

Life form traits responded to water quality improvements among all reaches (as indicated by relatively high variation explained by the covariate means of logN, logP, TSS, and chlorophyll; Figure 5). Submersed species had low occurrences in years with greater concentrations of logN, TSS, and chlorophyll, and submersed species increased in later years as water quality improved (Figure S3). The submersed species were positively associated with plots with higher hydrogeomorphic connectivity and exceedance values. The free‐floating plants were positively associated with spatial variations of water depth, connectivity, and logP. The entomophilous and epihydrophilous plants responded negatively during years of greater logP. Epihydrophilous plants favored high hydrogeomorphic connectivity, in support of our hypothesis.

The macrophyte communities within all four reaches were highly responsive both spatially and temporally to the river environment as evidenced by the model's *β* parameters (Figure 6, Figure S4) and marginal mean effects (Figure 7), indicating strong signals of environmental filtering and niche partitioning. The environmental responses varied by reach and species, but we found macroscale generalities across the four reaches that we summarize next:

**FIGURE 6:**
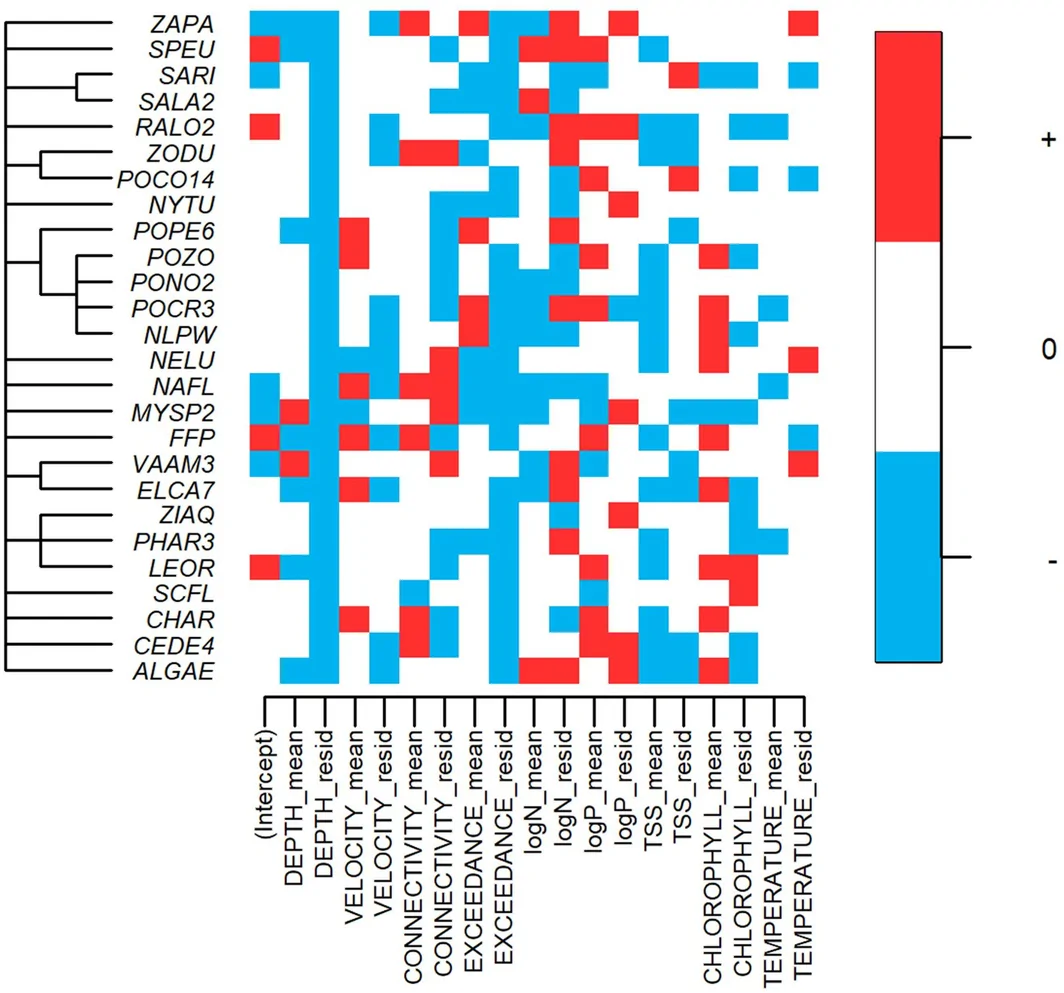
Diagram showing the relationships among macrophyte taxa (sorted as a taxonomic tree on the left) and their taxonomically‐structured responses to environmental covariates representing temporal and spatial variation in hydrology, water quality, and climate in Lower Pool 4 of the UMR. Positive and negative responses to each covariate (> 90% posterior probability) are illustrated in red and blue, respectively. Annual covariate means for Lower Pool 4 (“COVARIATE_mean”) represented the temporal variation in each environmental covariate across 22 years, and their within‐year residuals (“COVARIATE_residual”) represented spatial variation in environmental conditions within years. The macrophyte species codes are further described in Table S1. Similar plots for the other study reaches are in Figure S4.

**FIGURE 7:**
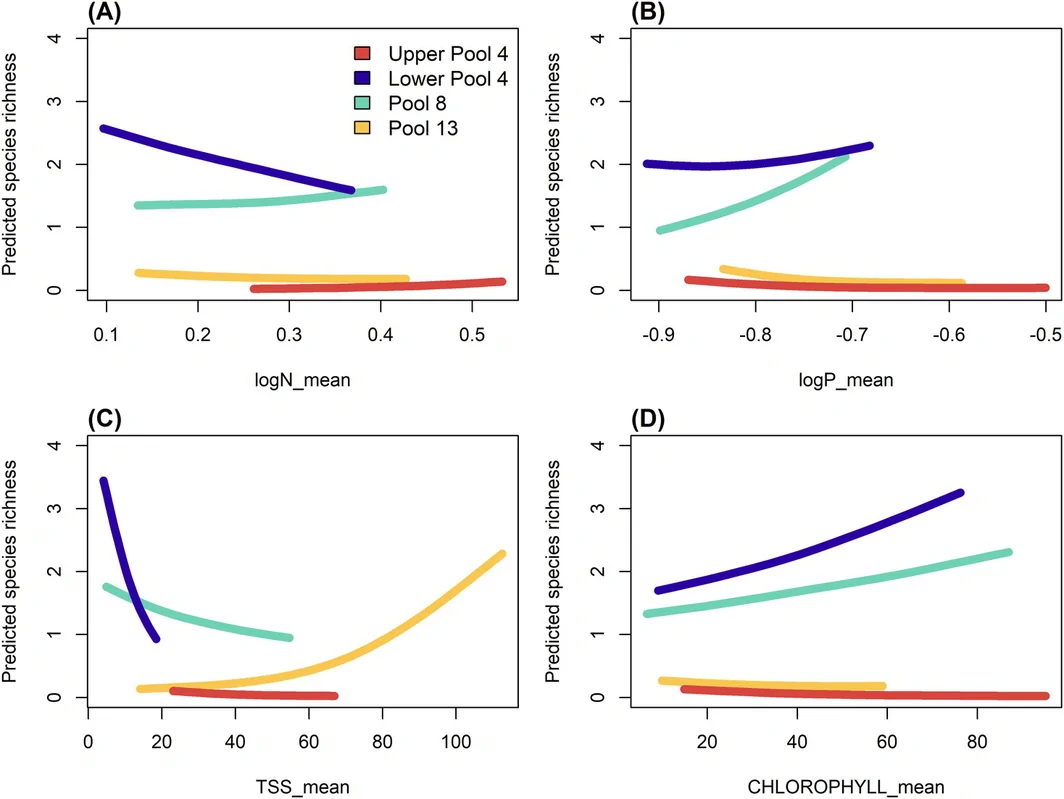
Marginal mean effects plots of the water quality covariates on macrophyte species richness in each study reach (differentiated by color). All covariates influenced richness either positively or negatively (> 90% posterior probability), although there was variability in magnitude and direction according to reach. The *x*‐axis values depict observed gradients in (A) total N concentration, log‐transformed (mg/L); (B) total P concentration, log‐transformed (mg/L); (C) total suspended solids (mg/L); and (D) chlorophyll *a* concentration (μg/L).

Water quality improvements had a pronounced effect on macrophyte richness as hypothesized, although there were notable differences among reaches (Figures 6 and 7). The two reaches with the greatest concentrations of logN, logP, TSS, and chlorophyll had very low species richness (Figure 7). Many species and taxonomic diversity were generally associated with productivity (i.e., greater concentrations of logP and chlorophyll), not supporting our hypothesis that the observed reductions of logP and chlorophyll would favor macrophytes. Although species richness and free‐floating plants increased with greater concentrations of chlorophyll and logP, most submersed species were negatively associated (Figures 5 and 6). Many species and biodiversity increased while TSS declined. More than 40% of species responded negatively to spatial and temporal increases in logN in all reaches (Figure S4). Species richness in Lower Pool 4 declined in years with higher average logN.

The complex hydrogeomorphology of the four river reaches and trends of increasing water levels were important for macrophyte distributions (Figure 6). Nearly all species in all reaches were negatively associated in space with greater water depth and velocity, which were the main spatial drivers. However, a few species were resilient to increasing water depth, perhaps explained by declines in TSS that allowed greater light penetration for photosynthesis. Species richness generally increased with greater exceedance and connectivity, which provides more shallow, wet habitats across the riverscape. As hypothesized, the increased hydrogeomorphic connectivity over time was favorable to many species, if the interplay between water depth and exceedance was optimal. The Mississippi River macrophytes preferred the hydrologically connected and shallow backwaters that these reaches provided during the study, particularly in later years with increased water in the ecosystem. Strong responses of macrophytes to hydrogeomorphic conditions in space and time reflect complex relationships with river bathymetry and changing water levels in the river and its floodplain.

Both common and rare species appeared to change with hydrogeomorphic changes and water quality improvements in all reaches (Figure 6). Species grouped as free‐floating plants changed with most environmental covariates that were modelled, especially with their temporal changes (> 90% posterior probability for covariate means). Taxa of particular interest to the UMR partnership for management and restoration, such as *Vallisneria americana* Michx. (VAAM3) and *Zizania* spp. (ZIAQ), were not well associated with included environmental covariates.

### 3.4 Species Co‐Occurrences at Multiple Spatial and Temporal Scales

In all reaches, we found patterns of macrophyte co‐occurrence that were stronger than expected by chance, even after accounting for environmental factors (Figure S5). At the plot‐level, most macrophyte species co‐occurred with positive associations except that five emergent species had strong negative associations with all other life forms. At the stratum‐level, residual associations were mostly positive. At the year‐level, species representing RCP1 and two invasive species ( *Potamogeton crispus* L. and *Myriophyllum spicatum* L.) had negative associations, which suggests these species had different temporal dynamics than other taxa.

## 4 Discussion

All the world's largest rivers have undergone various human disturbances during the Anthropocene with interactive effects on hydrogeomorphology, water quality, and climate (Datry et al. 2018; Huang et al. 2019; Sparks et al. 1998). Several large rivers recently experienced rather surprising, altered trajectories of macrophyte recovery, although the environmental drivers are not well documented or understood (Ibáñez et al. 2023; Larson et al. 2022). Here, we combine many UMR long‐term datasets and Bayesian joint species distribution models to parse the relative roles of spatial and temporal effects of water quality, hydrogeomorphology, and climate on a recovering riverine macrophyte community. All spatial hydrogeomorphic covariates were key environmental drivers, indicating the importance of spatial heterogeneity and water availability on macrophyte assembly in the river. The temporal changes to hydrogeomorphology reflect a wetter Mississippi River over several decades (Van Appledorn et al. 2026), and more water in the UMR has converted forest and agricultural areas into marsh and inundated, aquatic habitats (De Jager and Rohweder 2025). The macrophyte community responded somewhat in line with our water quality improvement (re‐oligotrophy) hypothesis, although the many exceptions and complexities are noteworthy. Macrophyte recovery and ecological trait shifts were well explained (up to 40% of variation) by water quality improvements. Despite that finding, all macrophyte species, ecological traits, and thus diversity were negatively affected by high TN. The residual association plots are consistent with species interactions like facilitation, which may have also fostered community recovery, or with shared environmental preferences not well captured in our models.

This unique dataset, to our knowledge, is the largest riverine macrophyte dataset to date. Our state‐of‐the‐art modelling approach with this big data lends to significant learning of major ecosystem changes that may be applied to inform the ecological restoration of other large rivers. Collectively, our results reveal the relative roles of spatial and temporal variations of hydrogeomorphology, water quality, and climate during two decades of macrophyte recovery in a large floodplain river.

### 4.1 Macrophyte Recovery in Relation to Water Quality Improvements

This is one of the first studies to model the associations of water quality improvements with riverine macrophyte recovery, although several other large rivers worldwide have experienced a similar re‐oligotrophy phenomenon (Diamond et al. 2022; Ibáñez et al. 2023). Conceptual frameworks predict similar outcomes to water quality compared to the results of this study (Dent et al. 2002; Hilton et al. 2006), but few papers show quantitative evidence because of a lack of data and the complexity of modelling such associations (Ibáñez et al. 2023). A long‐term study of macrophyte recovery in the Loire River of France found a decadal lag in shifting water quality and macrophyte responses (Diamond et al. 2022); however, here we show that Mississippi River macrophyte recovery occurred in less than a decade. Further, Davis et al. (2025) showed that water quality improvements in these UMR reaches occurred primarily over 6 years, which this study showed correspondence to a macrophyte regime shift around the same years of 2007–2013.

The effects and implications of water quality improvements and restoration can extend beyond macrophyte communities to other taxa of conservation concern and ecosystem processing rates. For instance, a presumed extinct freshwater fish was rediscovered after water quality improvements in Lake Constance of central Europe (Doenz and Seehausen 2020). Further, a shift from phytoplankton dominance to macrophyte dominance was linked to altered element cycling efficiency and net carbon balance in the Loire River of France (Diamond et al. 2022). Lastly, water quality improvements and macrophyte recovery have altered the diversity and ecological functions of macroinvertebrate and fish communities in rivers (Burdis et al. 2020; Tison‐Rosebery et al. 2022). The shift to macrophyte dominance in the UMR has affected certain fish communities and likely multiple ecosystem processes (Burdis et al. 2020; Houser 2022).

### 4.2 Continually Increasing Total Nitrogen Negatively Affected Nearly All Species and Traits

In rivers worldwide, the reduction of TP along with concomitant increases of TN affects the TN:TP ratio and has negative implications for primary production (Feio et al. 2023). The TN effect on UMR macrophytes may be ecologically significant because the HMSC models (which used presence/absence data) showed that localized species extinctions occurred in plots having high concentrations of TN. In this study, the decreases in TP partially contributed to macrophyte community recovery, and yet nearly all the species and diversity were also hindered by high loading and increasing TN. The TN and TP concentrations are often high throughout the Mississippi River and its tributaries (> 2.5 mg/L‐N), typically exceeding water quality standards in all years, and TN has increased over time (Jankowski 2022). Mississippi River TN loads may remain high given legacy N storage pools and the major basin‐wide changes required to lower TN (Jankowski 2022), as well as increasing UMR discharge that may concomitantly increase TN (Houser and Richardson 2010). Reducing TN in the Mississippi River is predicted to reduce vulnerability and increase resilience of submersed species (Delaney and Larson 2024), a finding reinforced by this study. Seemingly, water quality improvements are an important process shaping macrophyte community assembly in the UMR, but full biodiversity potential may be impeded by high TN.

### 4.3 The Role of Climate on Community Assembly

Our results suggest that direct effects of climate (in terms of water temperature only) played a minor role in macrophyte community assembly compared to other environmental drivers. However, hydrogeomorphology may to some extent be indirectly governed by climate via precipitation amounts or timing, and so the interactive effects of hydrogeomorphology and climate are difficult to disentangle from the models. Yet, many species were negatively associated with the spatial variation of higher water temperatures within reaches, with the warmest waters in Pool 13 that had a 1.5°C increase in average temperature over the past two decades. Unlike many terrestrial plants, aquatic macrophytes are thought to be less responsive to climate compared to local drivers like water quality and hydrogeomorphology (Alahuhta et al. 2021).

### 4.4 Inferring Species Interactions During the Recovery Period

The model's residual associations may suggest some localized plant species interactions or biophysical feedback loops that may have encouraged recovery alongside water quality improvements and hydrogeomorphic changes. Biofeedback loop mechanisms are suspected of influencing macrophyte recovery in the UMR (Burdis et al. 2020; Houser 2022). First, the mixture of positive and negative interspecific residual correlations indicates environmental niche differentiation among species. All the emergent species were negatively associated with other species and life forms, which may increase plant coverage in very shallow water that is unsuitable for submersed species (Larson, Carhart, and Lund 2023). Secondly, the universally positive residual associations among all submersed species at the plot‐level may reflect evidence of biofeedback and facilitation, whereby submersed species benefit from co‐occurrence with other species (Ovaskainen and Abrego 2020). Macrophytes can create localized, beneficial habitat conditions that may provide refuge from physical stresses like high velocity, improved resource availability from sediment‐nutrient recycling, and even spur further water quality improvements through nutrient uptake and improved water clarity. River restorations that can establish a few pollution‐tolerant species may facilitate new habitats that promote a greater abundance and diversity of macrophytes (Larson, Carhart, and Lund 2023). Also, restoration actions that maintain a short distance (< 1000 m) to nearby macrophyte beds are key to macrophyte resilience, likely by promoting dispersal and pollination (Carhart and De Jager 2019; Delaney and Larson 2024).

### 4.5 Potential Model Shortcomings and Opportunities to Advance Modelling

Our models were informative for addressing our hypotheses; however, the fractions of structured and unstructured random variations were often large, indicating substantial unexplained variance and noise in the ecological data. The explanatory powers of the models were modest, indicating that covariates may be missing and that the ecological processes of community assembly are complex, stochastic, and difficult to comprehensively model. The low predictive powers of the models indicate high variability and unpredictability of the river ecosystem, so caution is advised for forecasting. The residual correlations among taxa at the plot‐level were mostly positive, which implies that the models may have missed key environmental drivers or are reflecting species interactions of facilitation. Nevertheless, these complex models using large datasets allowed us to showcase the phenomenon of macrophyte assembly in relation to water quality improvements from multiple perspectives as intended.

It is also important to acknowledge that HMSC's parameter estimates may be biased when nontrivial proportions of occupied plots have low detection, a low probability of local occupancy, or both (Gray 2021). In such conditions, the effects of covariates on occupancy may be confounded by probabilities of detection and local occupancy. A new framework for incorporating imperfect detection into joint species distribution models is provided by Doser et al. (2023), but our current HMSC software did not allow for accurate detection estimates. Our field sampling has moderately high detection probabilities for common species (Gray 2021); therefore, the main results and conclusions in this study are not likely to have been qualitatively affected by detection probability.

Possibly, future HMSC models that can use plant species relative abundances (instead of simply presence/absence) would further clarify species‐environmental relationships. We did not model species abundances because the species were measured on an ordinal abundance scale that HMSC could not accommodate (Ovaskainen and Abrego 2020). Regardless, modeling species occurrences herein showed that macrophyte signals of environmental changes have been strong in the UMR. As examples, new species occurred following water quality improvements, and many species were completely absent within plots that had high TN.

### 4.6 The Novelty of Parsing Spatial and Temporal Variations

The approach we have taken here to parse the spatial and temporal variation in environmental conditions based on calculating annual covariate residuals and means was a methodological novelty with new ecological insights. First, this allowed us to address the relative roles of spatial and temporal environmental drivers, which are not well understood to date for macrophytes (Alahuhta et al. 2021). Secondly, the annual means of the environmental covariates revealed evidence of directional trends in species responses over time (including water quality improvements), allowing hypothesis testing. This new approach reveals that spatial heterogeneity within the large riverscape played a dominant role in shaping macrophyte communities, while also underscoring directional shifts that were associated with reach‐wide changes in environmental conditions, including water quality improvements (Jankowski 2022) and greater water volume (Van Appledorn et al. 2026) over time.

### 4.7 River Restoration and Management Applications

The UMR is so large that environmental restoration goals and tactics for water quality and macrophytes differ among its reaches (McCain et al. 2018). The reach‐level models identified generalities of regional patterns and processes to aid river restoration and management applications, but we sometimes found the four study reaches varied in their direction and magnitude of environmental associations. For example, unlike the other three study reaches, the community in Pool 13 was largely shaped by hydrogeomorphology and positively associated with TSS. As another example, the macrophyte communities of Lower Pool 4 were more strongly shaped by water quality than the other reaches, perhaps because the greatest environmental changes occurred in Lower Pool 4. When managing for specific macrophyte community types in this river (Larson, Bungula, et al. 2023; Larson, Carhart, and Lund 2023) or RCPs (in this study), consideration of the spatiotemporal context, river reach, and species‐specific environmental drivers is important.

Riverine macrophyte restoration may focus on the key controlling variables of flow rate, retention time, dilution, and water column nutrient inputs (Delaney and Larson 2024; Hilton et al. 2006) as represented by covariates in our HMSC models. In the UMR, there is opportunity to manipulate the environmental drivers in areas depauperate of aquatic plants (Carhart et al. 2021; Delaney and Larson 2024). For example, engineering features can increase hydrogeomorphic connectivity and water depth that reduce retention time and eutrophication, thus reducing free‐floating plants and improving submersed plant habitat (Heglund et al. 2022). In addition, catchment‐wide best management practices for nutrient reduction are still progressing in this basin, with more emphasis on reductions to TN and TSS than in previous decades (Upper Mississippi River Basin Association 2026).

Not all people celebrate the resurgence of macrophytes. Some scholars have long regarded native riverine plants as a nuisance for recreation or a source of economic and ecological harm (e.g., Holm et al. 1969). By contrast, surveys following a New Zealand riverine macrophyte restoration project found positive public views (Larned et al. 2006). So, when does native aquatic vegetation recovery alter human perceptions from a restoration success story to a serious problem warranting costly mechanical or chemical removal? In the UMR, this question has received little discussion, though the river management partnership has generally prioritized furthering macrophyte restoration (McCain et al. 2018). Still, some UMR river users have voiced concern that “excess native weeds” are unnatural, harms fisheries, and accelerate sedimentation. How many macrophytes constitute a desirable amount in the UMR remains open to debate. Regardless, our study demonstrates a clear ecological shift from a system dominated by undesirable free‐floating plants and algae to one supporting a more diverse, native assemblage of submersed and emersed vegetation.

## 5 Conclusions

We found macrophyte recovery was strongly associated with natural and spontaneous processes, such as increased water throughout the system, as well as human intervention through water policy and active habitat restorations through the UMRR program. Although human intervention remains essential for large river recovery, the resurgence of water quality and macrophytes can occur within a surprisingly short timeframe, on the order of less than a decade. Yet, as in many large ecosystems, the historical baseline for water quality and macrophyte composition in the UMR is unknown, and recovery may not simply mirror a linear reversal of past degradation. Therefore, ecosystems recovering from eutrophication and long‐term disturbance may not be fully reversible and are likely to experience shifting baselines (Duarte et al. 2009). This underscores the importance of setting realistic restoration targets and the need to conserve macrophyte communities that remain in quality condition today.

## Author Contributions

**Danelle M. Larson:** conceptualization, investigation, funding acquisition, writing – original draft, visualization, software, formal analysis, data curation, methodology, validation, project administration. **Mirkka M. Jones:** conceptualization, investigation, validation, methodology, visualization, writing – original draft, writing – review and editing, formal analysis, data curation. **Benjamin Weigel:** conceptualization, investigation, methodology, validation, visualization, writing – original draft, writing – review and editing, formal analysis. **Brian Gray:** conceptualization, investigation, methodology, validation, writing – review and editing. **Otso Ovaskainen:** conceptualization, investigation, methodology, validation, writing – review and editing, visualization, supervision.

## Funding

All data collection, the work of Danelle Larson, and the “2021 macrophyte restoration workshop” were funded through the US Army Corps of Engineers' Upper Mississippi River Restoration program Long Term Resource Monitoring (LTRM) element. The work of Mirkka Jones was supported by the Research Council of Finland's “Thriving Nature” research profiling action as a member of the University of Helsinki Biodata Analytics Unit. The work of Benjamin Weigel was supported by the Strategic Research Council and the Academy of Finland (Project 312650 BlueAdapt). The work of Otso Ovaskainen was funded by Academy of Finland (grant no. 336212 and 345110) and the European Union: The European Research Council (ERC) under the European Union's Horizon 2020 research and innovation programme (grant agreement no. 856506: ERC‐synergy project LIFEPLAN). The work of Brian Gray was contributed following retirement from the US Geological Survey.

## Conflicts of Interest

The authors declare no conflicts of interest.

## Supporting information


**Figure S1:** Figure showing scaled (−1 to 1) prevalence of macrophyte species from all four reaches of the UMR within two regions of common profile (RCP1 and RCP2) as defined by a *k*‐means clustering algorithm. Darker shades indicate relatively higher prevalences of the species. Full species information is found in Table S1.
**Figure S2:** Temporal shifts for the RCP of four study reaches of the UMR from years 1998 to 2019. The RCP were defined on species composition of 35 aquatic macrophytes. Species composition for RCP1 and RCP2 is in Figure S1.
**Figure S3:** Predicted macrophyte trait responses to water quality from four study reaches (Pool) of the UMR. The traits shown are life forms as submersed plants (red) or free‐floating plants (blue). All covariates shown have > 90% posterior probability that the life form distributions are influenced by the water quality covariates of (A) chlorophyll *a* concentration (μg/L); (B) total nitrogen concentration, log‐transformed (mg/L); (C) total phosphorus concentration, log‐transformed (mg/L); and (D) total suspended solids (mg/L).
**Figure S4:** The relationships among macrophyte taxa (taxonomic tree on the left) and their taxonomically‐structured responses to environmental covariates representing temporal and spatial variation in hydrology, water quality, and climate in four reaches of the UMR (Panels 3a–d). Positive and negative responses (> 90% posterior probability) are illustrated by the red and blue colors, respectively. Annual covariate means per reach (“COVARIATE_mean”) represented the temporal variation in each environmental covariate across 22 years and their within‐year residuals (“COVARIATE_resid”) represented spatial variation in environmental conditions within years. The macrophyte species codes on the phylogenetic tree are standards from the USDA Plants Database and are further described in Table S1.
**Figure S5:** Residual associations among 26 macrophyte species at three spatiotemporal scales within Lower Pool 4, UMR. The macrophyte species codes are standards from the USDA Plants Database and further described in Table S1. The associations are graphed for the plot‐level (A), stratum‐level (B), and year‐level (C). Pairs of species illustrated by red and blue show positive or negative associations, respectively (> 90% posterior probability). Residual associations may be interpreted as species co‐occurrences after accounting for their environmental niches.
**Table S1:** Aquatic macrophyte species found throughout the UMR. Data were retrieved on 20 March 2021 from https://umesc.usgs.gov/data_library/vegetation/srs/veg_srs_1_query.shtml. We list 71 aquatic macrophyte species over the period of record (1998–2023) in the UMR. Species' codes are from the USDA Plants Database, https://plants.sc.egov.usda.gov/home and taxonomic authority was used from the Integrated Taxonomic Information System (ITIS) in 2024. Life form includes submersed (“S”), emersed (“E”), rooted floating‐leaved (“F”), nonrooted floating‐leaved (“N”), and filamentous algae (“A”). Pollination mode includes entomophily (by insects), hypohydrophily (under the water's surface), epihydrophily (on the water's surface), or N/A (not available because algae does not pollinate). Native status includes either native (“N”) or not native/invasive (“NN/I”) to the UMR Basin. USDA Wetland status includes obligate wetland (“OBL”) and facultative wetland (“FACW”) for the Midwest Region of the United States. Species are listed in order of most common occurrences as of 2019.
**Table S2:** The average proportion of variation in macrophyte species occurrences explained by each fixed effect environmental covariate as determined by variance partitioning. Covariates were modeled separately for each reach's annual mean (“COVARIATE_mean”), which indicates temporal variation through 22 years, and the annual residual (“COVARIATE_residual”), which indicates the spatial variation within years. Covariates are grouped by gray shading of rows.
**Table S3:** For each of four UMR river reaches, the percentage of variation in each macrophyte species' occurrences explained by each fixed effect environmental covariates determined by variance partitioning. Covariates were modeled separately as each reach's annual mean, which indicates temporal variation through 22 years, and annual residual, which indicates spatial variation.

## Data Availability

All original data are publicly available through the Upper Mississippi River Restoration Program's Long Term Resource Monitoring (https://umesc.usgs.gov/ltrm‐home.html) and the National Water Information System (https://waterdata.usgs.gov/nwis). Our secondary data are permanently archived on ScienceBase (https://doi.org/10.5066/P1JGFM76). The data analysis scripts and secondary datasets are also on Zenodo (https://doi.org/10.5281/zenodo.22283226).
