# Supplementary material for: Water Quality Improvements and Hydrogeomorphic Change Over 20 Years Contributed to Macrophyte Community Recovery in the Upper Mississippi River
Source: Glob Chang Biol. 2026 Oct 6;32(10):e71121. doi: 10.1111/gcb.71121 (PMC13639705; doi:10.1111/gcb.71121)
Supplement: Supplementary file 1 [file GCB-32-e71121-s001.docx]

**Supplemental Materials for:**

**Water Quality Improvements and Hydrogeomorphic Change Over 20 Years Contributed to Macrophyte Community Recovery in the Upper Mississippi River**

Danelle M. Larson,

-U.S. Geological Survey, 2630 Fanta Reed Road, La Crosse, WI 54603;

e-mail: [dmlarson@usgs.gov](mailto:dmlarson@usgs.gov); [danellelarson88@gmail.com](mailto:danellelarson88@gmail.com); 608-781-6350

ORCID: 0000-0001-6349-6267

*corresponding author

Mirkka M. Jones,

-Research Centre for Ecological Change, Organismal and Evolutionary Biology Research Programme, University of Helsinki, Helsinki, Finland

-Institute of Biotechnology, HILIFE Helsinki Institute of Life Science, University of Helsinki, Helsinki, Finland

e-mail: mirkka.jones@gmail.com

ORCID: 0000-0002-8157-8730

Benjamin Weigel,

- INRAE, EABX, 33612 Cestas, France

- Research Centre for Ecological Change, Organismal and Evolutionary Biology Research Programme, University of Helsinki, Helsinki, Finland

e-mail: benjamin.weigel@inrae.fr

ORCID: 0000-0003-2302-5529

Brian Gray,

-U.S. Geological Survey, 2630 Fanta Reed Road, La Crosse, WI 54603;

e-mail: [brgray@usgs.gov](mailto:brgray@usgs.gov)

ORCID: 0000-0001-7682-9550

Otso Ovaskainen,

-Department of Biological and Environmental Science, University of Jyväskylä, Jyväskylä, Finland

e-mail: [otso.t.ovaskainen@jyu.fi](mailto:otso.t.ovaskainen@jyu.fi)

ORCID: 0000-0001-9750-4421

**Publication Journal:** Global Change Biology; article DOI : 10.1111/gcb.71121

**SUPPLEMENTAL TABLE 1** Aquatic macrophyte species found throughout the UMR. Data were retrieved on 20 March 2021 from <https://umesc.usgs.gov/data_library/vegetation/srs/veg_srs_1_query.shtml>. We list 71 aquatic macrophyte species over the period of record (1998–2023) in the UMR. Species’ codes are from the USDA Plants Database, and taxonomic authority was used from the Integrated Taxonomic Information System (ITIS) in 2024. Life form includes submersed (“S”), emersed (“E”), rooted floating-leaved (“F”), nonrooted floating-leaved (“N”), and filamentous algae (“A”). Pollination mode includes entomophily (by insects), hypohydrophily (under the water’s surface), epihydrophily (on the water’s surface), anemophilous (pollinated by wind), or N/A (not available because algae does not pollinate). Native status includes either native (“N”) or not native/invasive (“NN/I”) to the UMR Basin. USDA Wetland status includes obligate wetland (“OBL”) and facultative wetland (“FACW”) for the Midwest Region of the United States. Species are listed in order of most common occurrences as of 2019.

| Species’ code | Common names | Family | Scientific name | Life form | Pollination mode | Native status | USDA wetland status (Midwest region) |
| --- | --- | --- | --- | --- | --- | --- | --- |
| CEDE4 | coontail | Ceratophyllaceae | *Ceratophyllum demersum* L. | S | Hypohydrophily | N | OBL |
| ELCA7 | Canadian waterweed | Hydrocharitaceae | *Elodea canadensis* Michx. | S | Epihydrophily | N | OBL |
| VAAM3 | watercelery, wild celery | Hydrocharitaceae | *Vallisneria americana* Michx. | S | Epihydrophily | N | OBL |
| LEMI3 (FFP) | small duckweed | Lemnaceae | *Lemna minor* L. | N | Entomophily | N | OBL |
| ZODU | water stargrass, grassleaf mudplantain | Pontederiaceae | *Heteranthera dubia* (Jacq.) MacM. | S | Epihydrophily | N | OBL |
| NLPW | narrow-leaf pondweed | Potamogetonaceae | *Potamogeton foliosus* Raf. or  *Potamogeton pusillus* L. | S | Epihydrophily | N | OBL |
| ALGA | filamentous algae (not speciated) | N/A | Filamentous algae | A | N/A | N | OBL |
| POPE6 | sago pondweed | Potamogetonaceae | *Stuckenia pectinata* (L.) Börner | S | Hypohydrophily | N | OBL |
| SPPO (FFP) | big duckweed | Lemnaceae | *Spirodela polyrrhiza* (L.) Schleid. | N | Entomophily | N | OBL |
| NYTU | white waterlily | Nymphaeaceae | *Nymphaea odorata* Aiton subsp. *tuberosa* (Paine) Wiersma & Hellquist | F | Entomophily | N | OBL |
| MYSP2 | Eurasian watermilfoil | Haloragaceae | *Myriophyllum spicatum* L. | S | Epihydrophily | NN/I | OBL |
| POCR3 | curly-leaved pondweed | Potamogetonaceae | *Potamogeton crispus* L. | S | Epihydrophily | NN/I | OBL |
| NELU | lotus, American lotus | Nelumbonaceae | *Nelumbo lutea* Willd. | F | Entomophily | N | OBL |
| POZO | flatstem pondweed | Potamogetonaceae | *Potamogeton zosteriformis* Fern. | S | Epihydrophily | N | OBL |
| WOCO  (FFP) | Columbian watermeal | Lemnaceae | *Wolffia columbiana* Karst. | N | Entomophily | N | OBL |
| SALA2 | broadleaf arrowhead | Alismataceae | *Sagittaria latifolia* Willd. | E | Entomophily | N | OBL |
| SARI | sessilefruit arrowhead | Alismataceae | *Sagittaria rigida* Pursh. | E | Entomophily | N | OBL |
| PONO2 | longleaf pondweed | Potamogetonaceae | *Potamogeton nodosus* Poir. | S | Epihydrophily | N | OBL |
| ZIAQ | wild rice | Poaceae | *Zizania spp.* (genetics unknown as of 2024) | E | Anemophily | N | OBL |
| NAFL | nodding waternymph | Najadaceae | *Najas flexilis* (Willd.) Rostk. & Schmidt | S | Hypohydrophily | N | OBL |
| LETR (FFP) | star duckweed | Lemnaceae | *Lemna trisulca* L. | N | Entomophily | N | OBL |
| SPEU | giant burreed | Sparganiaceae | *Sparganium eurycarpum* Engelm. ex Gray | E | Anemophily | N | OBL |
| SCFL | river bulrush | Cyperaceae | *Bolboschoenus fluviatilis* (Torr.) Soják | E | Anemophily | N | OBL |
| PHAR3 | reed canarygrass | Poaceae | *Phalaris arundinacea* L. | E | Anemophily | NN/I | FACW |
| NAGU | southern waternymph | Najadaceae | *Najas guadalupensis* (Spreng.) Magnus | S | Hypohydrophily | N | OBL |
| UTMA | common bladderwort | Lentibulariaceae | *Utricularia vulgaris* Le Conte subsp. macrorhiza | S | Entomophily | N | OBL |
| RALO2 | longbeak buttercup | Ranunculaceae | *Ranunculus aquatilis* var. diffusus | S | Entomophily | N | OBL |
| LEOR | rice cutgrass | Poaceae | *Leersia oryzoides* (L.) Sw. | E | Anemophily | N | OBL |
| MYSI | northern watermilfoil | Haloragaceae | *Myriophyllum sibiricum* Komarov | S | Epihydrophily | N | OBL |
| CHAR | chara (macro-algae), muskgrass | Characeae | *Chara* spp. | S | N/A | N | OBL |
| NAMI | brittle naiad | Hydrocharitaceae | Najas minor All. | S | Hypohydrophily | NN/I | OBL |
| SCVA | softstem bulrush | Cyperaceae | *Schoenoplectus tabernaemontani* (K.C. Gmel.) Palla | E | Anemophily | N | OBL |
| POCO14 | pickerelweed | Pontederiaceae | *Pontederia cordata* L. | E | Entomophily | N | OBL |
| ZAPA | horned pondweed | Zannichelliaceae | *Zannichellia palustris* L. | S | Hypohydrophily | N | OBL |
| NULU | yellow pond lily | Nymphaeaceae | *Nuphar lutea* (L.) Sm. | F | Entomophily | N | OBL |
| PORI2 | Richardson's pondweed | Potamogetonaceae | *Potamogeton richardsonii* (Benn.) Rydb. | S | Epihydrophily | N | OBL |
| BUUM | flowering rush | Butomaceae | *Butomus umbellatus* L. | E | Entomophily | NN/I | FACW/OBL |
| ECCR | barnyard grass | Poaceae | *Echinochloa crus-galli* (L.) P. Beauv. | E | Anemophily | NN/I | OBL |
| LYSA2 | purple loosestrife | Lythraceae | *Lythrum salicaria* L. | E | Entomophily | NN/I | FACW/OBL |
| POAM8 | water knotweed | Polygonaceae | *Polygonum amphibium* L. | E | Entomophily | N | OBL |
| POHY2 | swamp smartweed | Polygonaceae | *Polygonum hydropiperoides* Michx. | E | Entomophily | N | OBL |
| SANI | black willow | Salicaceae | *Salix nigra* Marshall | E | Entomophily | N | FACW/OBL |
| TYAN | narrowleaf cattail | Typhaceae | *Typha angustifolia* L. | E | Aneomophily | NN/I | OBL |
| TYLA | broadleaf cattail | Typhaceae | *Typha latifolia* L. | E | Anemophily | N | OBL |

**SUPPLEMENTAL TABLE 2** The average proportion of variation in macrophyte species occurrences explained by each fixed effect environmental covariate as determined by variance partitioning. Covariates were modeled separately for each reach’s annual mean (“COVARIATE_mean”), which indicates temporal variation through 22 years, and the annual residual (“COVARIATE_residual”), which indicates the spatial variation within years. Covariates are grouped by gray shading of rows.

| **Covariate grouping** | **Covariate** |  | **Proportion of variation explained** |  |  |
| --- | --- | --- | --- | --- | --- |
|  |  |  |  |  |  |
|  |  |  |  |  |  |
|  |  | **Upper Pool 4** | **Lower Pool 4** | **Pool 8** | **Pool 13** |
|  | (Intercept) | 0.54 | 0.06 | 0.14 | 0.14 |
| Hydrogeomorphology | AVGDEPTH_mean | 0.13 | 0.14 | 0.08 | 0.08 |
| Hydrogeomorphology | AVGDEPTH _residual | 0.56 | 0.13 | 0.07 | 0.07 |
| Hydrogeomorphology | VELOCITY_mean | 0.58 | 0.08 | 0.16 | 0.14 |
| Hydrogeomorphology | VELOCITY _ residual | 0.23 | 0.16 | 0.19 | 0.18 |
| Hydrogeomorphology | CONNECTIVITY_ mean | 0.42 | 0.12 | 0.11 | 0.11 |
| Hydrogeomorphology | CONNECTIVITY _ residual | 0.1 | 0.12 | 0.21 | 0.22 |
| Hydrogeomorphology | EXCEEDANCE_ mean | 0.25 | 0.12 | 0.08 | 0.08 |
| Hydrogeomorphology | EXCEEDANCE _ residual | 0.15 | 0.34 | 0.06 | 0.06 |
| Water quality | logN_ mean | 0.41 | 0.31 | 0.04 | 0.04 |
| Water quality | logN_ residual | 0.21 | 0.06 | 0.14 | 0.14 |
| Water quality | logP_ mean | 0.66 | 0.05 | 0.04 | 0.04 |
| Water quality | logP_ residual | 0.1 | 0.06 | 0.05 | 0.05 |
| Water quality | TSS_ mean | 0.27 | 0.08 | 0.28 | 0.28 |
| Water quality | TSS_ residual | 0.19 | 0.12 | 0.13 | 0.13 |
| Water quality | CHLOROPHYLL_ mean | 0.51 | 0.12 | 0.13 | 0.13 |
| Water quality | CHLOROPHYLL _ residual | 0.14 | 0.03 | 0.09 | 0.08 |
| Climate | TEMPERATURE_ mean | 0.14 | 0.07 | 0.16 | 0.16 |
| Climate | TEMPERATURE _ residual | 0.2 | 0.12 | 0.16 | 0.16 |

**SUPPLEMENTAL TABLE 3** For each of four UMR river reaches, the percentage of variation in each macrophyte species’ occurrences explained by each fixed effect environmental covariates determined by variance partitioning. Covariates were modeled separately as each reach’s annual mean, which indicates temporal variation through 22 years, and annual residual, which indicates spatial variation.

**SUPPLEMENTAL FIGURE 1** Figure showing scaled (−1 to 1) prevalence of macrophyte species from all four reaches of the UMR within two regions of common profile (RCP1 and RCP2) as defined by a *k-means* clustering algorithm. Darker shades indicate relatively higher prevalences of the species. Full species information is found in Supplemental Table 1.

**SUPPLEMENTAL FIGURE 2** Temporal shifts for the RCP of four study reaches of the UMR from years 1998–2019. The RCP were defined on species composition of 35 aquatic macrophytes. Species composition for RCP1and RCP2 is in Supplemental Figure 1.

**SUPPLEMENTAL FIGURE 3** Predicted macrophyte trait responses to water quality from four study reaches (Pool) of the UMR. The traits shown are life forms as submersed plants (red) or free-floating plants (blue). All covariates shown have >90% posterior probability that the life form distributions are influenced by the water quality covariates of (A) chlorophyll *a* concentration (µg/L); (B) total nitrogen concentration, log-transformed (mg/L); (C) total phosphorus concentration, log-transformed (mg/L); and (D) total suspended solids (mg/L).

4a – Upper Pool 4

4b – Lower Pool 4

4c – Pool 8

4d – Pool 13

**SUPPLEMENTAL FIGURE 4** The relationships among macrophyte taxa (taxonomic tree on the left) and their taxonomically-structured responses to environmental covariates representing temporal and spatial variation in hydrology, water quality, and climate in four reaches of the UMR (Panels 3a–d). Positive and negative responses (>90% posterior probability) are illustrated by the red and blue colors, respectively. Annual covariate means per reach (“COVARIATE_mean”) represented the temporal variation in each environmental covariate across 22 years and their within-year residuals (“COVARIATE_resid”) represented spatial variation in environmental conditions within years. The macrophyte species codes on the phylogenetic tree are standards from the USDA Plants Database and are further described in Supplemental Table 1.

**SUPPLEMENTAL FIGURE 5** Residual associations among 26 macrophyte species at three spatiotemporal scales within Lower Pool 4, UMR. The macrophyte species codes are standards from the USDA Plants Database and further described in Supplemental Table 1. The associations are graphed for the plot-level (A), stratum-level (B), and year-level (C). Pairs of species illustrated by red and blue show positive or negative associations, respectively (>90% posterior probability). Residual associations may be interpreted as species co-occurrences after accounting for their environmental niches.
